# Effects of Antipsychotics on Dentate Gyrus Stem Cell Proliferation and Survival in Animal Models: A Critical Update

**DOI:** 10.1155/2012/832757

**Published:** 2012-10-24

**Authors:** Gerburg Keilhoff, Paolo Fusar-Poli, Axel Becker

**Affiliations:** ^1^Institute of Biochemistry and Cell Biology, University of Magdeburg, Leipziger Straße 44, 39120 Magdeburg, Germany; ^2^Institute of Psychiatry, King's College London, 5th Floor, Main Building, P.O. Box 63, De Crespigny Park, London SE5 8AF, UK; ^3^Institute of Pharmacology and Toxicology, University of Magdeburg, Leipziger Straße 44, 39120 Magdeburg, Germany

## Abstract

Schizophrenia is a complex psychiatric disorder. Although a number of different hypotheses have been developed to explain its aetiopathogenesis, we are far from understanding it. There is clinical and experimental evidence indicating that neurodevelopmental factors play a major role. Disturbances in neurodevelopment might result in alterations of neuroanatomy and neurochemistry, leading to the typical symptoms observed in schizophrenia. The present paper will critically address the neurodevelopmental models underlying schizophrenia by discussing the effects of typical and atypical antipsychotics in animal models. We will specifically discuss the vitamin D deficiency model, the poly I:C model, the ketamine model, and the postnatal ventral hippocampal lesion model, all of which reflect core neurodevelopmental issues underlying schizophrenia onset.

## 1. Introduction

Schizophrenia is a complex psychiatric disorder which is characterized by a defined set of symptoms usually grouped into positive symptoms, negative symptoms, cognitive impairment, psychosocial impairments, and poor quality of life. It is commonly described as a developmental disorder, with onset in the early adulthood or adolescence and involving several genetic and environmental factors. The causes of schizophrenia are unknown. However, several hypotheses have been tested in the recent research. One of the most accepted theories is the *“two hit hypothesis.”* Such a hypothesis proposes that an early disturbance is necessary but not sufficient to cause an increased vulnerability to schizophrenia. Thus, an early neurodevelopmental insult is requested to interact with either normal or abnormal postpubertal brain maturation to fully produce late neurodevelopmental brain structural and functional changes [1–4].

Evidence indicating a neurodevelopmental origin of schizophrenia is grounded on extensive research performed over the past two decades. In particular, a novel approach has allowed clinicians to specifically investigate the early phases of psychosis and to clarify the mechanisms underlying the onset of the illness. This approach has been variably termed as ultrahigh risk, at risk mental state, or clinical high risk [5]. This putatively prodromal psychotic phase is associated with an enhanced risk of developing the illness as compared to the general population (1%), ranging from 18% at six months up to 36% after three years [6]. The majority (73%) of the individuals developing a psychotic illness will transit towards a schizophrenia spectrum disorder [7]. The clinical high risk state for psychosis is also characterized by significant cognitive impairments [8] and deficits in social functioning and quality of life [5]. These alterations are associated with underlying neurodevelopmental abnormalities in the structure [9–11], function [12–14], connectivity [15], and neurochemistry [16–18] of the brain, resembling those observed in the established phase of the illness [19]. Interest in this area has exponentially grown to the extent that a new diagnostic category is being discussed in the forthcoming DSM-5 [20]. These findings taken together indicated that schizophrenia is characterized by dynamic neurobiological changes since its earliest phases. In theory, the early phases of schizophrenia can thus be particularly amenable to treatments that can impact the underlying neurobiology, including antipsychotics. The present paper will critically address this point, focusing on the role played by the effect of antipsychotics on the neurogenesis during the onset of schizophrenia. These issues will be discussed in the light of the recent advances in animal models.

## 2. Methodological Approach 

In the following sections, we will provide an update on the effects of antipsychotics on cell proliferation in animal models used in schizophrenia research. This critical paper is limited to models most traditionally employed in the laboratories, whereby all models reflect neurodevelopmental aspects. In Table 1, a selection of most relevant publications in the last 13 years is given. A survey of effects of neuroleptics on hippocampal neurogenesis is provided in Table 2.

## 3. Modelling Schizophrenia with Animal Paradigms

The development of animal models is a crucial issue in biological psychiatry for the study of alterations in neurochemistry, neuroanatomy, and behaviours resembling those observed in schizophrenia. Similarly, it can be useful for the discovery and development of effective treatments such as antipsychotic molecules. However, heterogeneity of the clinical symptoms of schizophrenia and the incomplete knowledge about the cause and progression of the illness make the development of valid animal models particularly difficult. Moreover, there is scepticism as to what extent the behaviour of animals can actually reflect highly complex disorders such as schizophrenia. Since each animal model is developed to target only each specific domain of schizophrenia, different complementary approaches are usually needed. Therefore (i) developmental, (ii) genetic, and (iii) pharmacological models have been used in experimental schizophrenia research [31–38].

(i) The developmental hypothesis and the respective animal models proceed from the assumption that malformations at very early stages of neurogenesis result in structural abnormalities of the adult brain [39–41]. Under this scenario, the pathogenesis of schizophrenia onset is attributed to abnormal neuronal development and/or re-organization of neuronal circuits in the frontal cortex or in limbic structures. Hippocampal volume reductions [42, 43], hippocampal shape deformation, or abnormalities in the hippocampal cell density [44] have been consistently reported. Many of these alterations, however, are essentially based on genetic deficits impacting the brain development (see below). 

(ii) Genetic animal models are developed by translation of human genetic mutations into animals (for review: [33, 45–47]). They include whole-body mutant mouse strains ([48] (Reelin); [49] (Neuregulin-1)), mutant mouse strains in which distinct genes have been knocked down in a tissue- or cell-type-specific manner ([50] (NRG-1/ErbB); [51] (DISC)), and transgenic mice that overexpress schizophrenia-relevant genes ([52] (dopamine D2 receptor); [53] (Neuregulin-1)). More advanced and complex models, however, are under development. These models combine several environmental and/or genetic factors to better account for the complex aetiology of schizophrenia [54]. For example, combined genetic disruption of the NMDA receptor subunit 1 [55–58], the dopamine D2 and D4 receptors, the dopamine transporter [59–61], and, the mutants in the dopamine-degrading enzyme catechol-O-methyl-transferase [62, 63] may provide a unique tool to study imbalance in the functional regulation of neurotransmitters implicated in schizophrenia.

(iii) Finally, the development of schizophrenia-relevant animal models can also target the pharmacodynamics of common antipsychotic drugs, to identify their molecular substrates, and to optimise their pharmacokinetics, to develop new drugs, or to test potential antipsychotics. 

## 4. Concept of Neurogenesis in Schizophrenia

Neuronal stem cells (NSCs) belong to the class of adult stem cells. They are multipotent and able to generate (only) the specific cell lineages of the nervous system: neurons, astrocytes, and oligodendrocytes [68]. NSCs will be generated throughout the whole life, but with declining intensity. NSCs are primarily located in the subgranular zone (SGZ) of the hippocampal dentate gyrus and in the subventricular zone (SVZ) of the lateral ventricles. But there is also evidence that NSCs are present in multiple areas of the adult brain [69]. Under the influence of their local microenvironment, that is, their niche, NSCs take different developmental pathways/roads of life. NSCs in the SVZ become neuroblasts, migrate towards the rostral migratory stream into the olfactory bulb, and develop into interneurons. NSCs in the SGZ, on the other hand, develop into local dentate granule cells [70]. 

Even if most evidences were acquired from different animal models, there is converging consensus that adult neurogenesis seems to be essential for different processes, such as learning and memory [71, 72], mood regulation [73, 74], physiological (maintenance) neuroregeneration, neurorestoration after mechanical brain injuries [75, 76], stroke [76, 77], multiple sclerosis [78, 79], and Parkinson's disease [80]. Thus it is not surprising that disturbances of adult neurogenesis are investigated in a wide range of pathological processes including neurodegenerative diseases, brain tumours, seizures, and mental illnesses such as schizophrenia, major depression, dementia, and alcoholism [70].

Because of these reasons, over the past years there has been a growing interest into neurogenesis-relevant research on postmortem human tissues of schizophrenic patients. Arnold and Watt [21] found abnormal neuronal densities in the olfactory epithelium of schizophrenics. Rioux and Arnold [81] demonstrated a deregulated expression of retinoid receptors in schizophrenia and that retinoid signalling plays a central role in neurogenesis. Reif and coworkers [22] were able to demonstrate that the first step of adult neurogenesis, that is, cell proliferation, is diminished in the dentate gyrus of patients suffering from schizophrenia. Atz and coworkers [82] found a genotypic association of NCAM1 polymorphisms with schizophrenia. NCAM expression is a characteristic feature of the postnatal neurogenic niche [83]. 

Beside differentiation into neurons, adult neuronal stem cells may also undergo gliogenesis. There is an increasing body of evidence for glial pathology in schizophrenia [84]. Oligodendrocyte and myelin dysfunction are perturbed in schizophrenia since its earliest phases. Through changes in synaptic formation and/or function, they can induce cognitive dysfunction, one of the core symptoms of schizophrenia [85, 86]. The activation of astrocytes has been discussed as an important pathogenic factor for the development of schizophrenia [87], and also microglia has been shown to remodel the CNS during development as well as after injuries [88–92].

## 5. Neurogenic Potential of Antipsychotics

Basically, antipsychotic drugs are divided into typical (first-generation antipsychotics) and atypical (second-generation antipsychotics). Within the commonly used typical antipsychotics in early psychosis are butyrophenone (e.g., haloperidol) and phenothiazine (e.g., chlorpromazine) derivates and within the atypical are clozapine, olanzapine, quetiapine, risperidone (reviewed in [93]). 

Antipsychotics interfere with neuronal remodelling. Thereby dopaminergic effects seem to be involved [23, 94]. It was suggested that stimulation of dopamine D2 receptors inhibits the proliferation of neuronal stem cells and that tonic endogenous dopamine inhibits their proliferation [88]. Moreover, it was reported that blockade of D2 receptors activates transcription factors which regulate the expression of genes of neuronal growth factors [95].

Olanzapine, which has less affinity for the dopaminergic receptors [96], is also able to enhance SVZ [24, 25] and hippocampal neurogenesis [26]. The latter, on the other hand, is not influenced by haloperidol [24, 27] clearly indicating that typical and atypical antipsychotics differentially regulate neurogenesis. In support to this notion, it was also reported that typical and atypical antipsychotics differentially induce neuronal plasticity and synaptic remodelling. Atypical but not typical antipsychotics are effective not only in the striatum but also in the prefrontal cortex and hippocampus [94]. With respect to the schizophrenia-relevant morphometric changes and cellular abnormalities in the grey matter, the reported effects of antipsychotics in the prefrontal cortex are particularly interesting (reviewed in [97]). Interestingly, long-term antipsychotic treatment induced glial but not neuronal cell proliferation in monkeys and that with no difference between typical and atypical antipsychotics [98]. All these results, however, do not challenge the scientific consensus that the adult cortex under physiological conditions belongs to the so-called nonneurogenic tissue. 

## 6. Mechanisms Underlying the Effects of Antipsychotics on Neurogenesis

There are different possible mechanisms by which antipsychotics realize their influence on cell proliferation/neurogenesis. Earlier studies indicate that a couple of factors, for example, trophic and transcription factors, can interact in a fine-tuned network. Clozapine, for example, selectively increased FGF-2 (fibroblast growth factor-2, belongs to trophic factors) in the striatum [99]. In the hippocampus, FGF-2 is induced by quetiapine, but only when the NMDA receptor system is downregulated [100]. Other studies addressed the schizophrenia-relevant role of BDNF (brain-derived neurotrophic factor). However, the findings on BDNF status in naïve patients as well as in patients treated with antipsychotics are highly discrepant (reviewed by [101–105]). In animals, BDNF in the hippocampus was decreased by haloperidol and high-dosed risperidone [106–108], while olanzapine therapy enhanced BDNF [109]. It was also shown that haloperidol reduced NGF (neuronal growth factor) while olanzapine raised NGF levels and risperidone was ineffective on NGF [110, 111]. VEGF (vascular endothelial growth factor, angiogenic neurotrophin) seemed also to be involved in the action of antipsychotics. Haloperidol and olanzapine increased its hippocampal levels [112]. 

Antipsychotics can additionally influence cell proliferation/neurogenesis via targeting transcription factors implicated in mitotic activity regulation. Thus, it was shown that haloperidol, risperidone, and clozapine affected phosphorylation of extracellular signal-regulated kinases (ERKs) and cyclic adenosine 3′,5′-monophosphate (cAMP) response element (CRE) binding protein (CREB), each with different profiles. In fact, haloperidol and risperidone promoted phosphorylation [113, 114], while clozapine reduced ERK1/2 and CREB phosphorylation [113]. Furthermore, haloperidol treatment of mice increased phosphorylation of Akt1. With respect to the Akt/GSK-3 system, clozapine had similar effects as haloperidol increasing the Akt1 phosphorylation [115].

It is important to note that a direct antipsychotic drug-gene interaction should be taken into close consideration, even if a direct intervention on genes (belonging to the glutamate/NMDA receptor family) has been shown only for haloperidol [116, 117]. Since modulation of progenitor cell proliferation as well as neurogenesis resulting in NMDA receptor modulation has been described [118], these findings set one possible agenda by which a direct antipsychotic drug-gene interaction can become neurogenic. 

Assuming that the pathology of the schizophrenia [119, 120] and the cell proliferation/neurogenesis [70, 121] are subjected to epigenetic control mechanisms, future research is needed to address the exact neurogenic mechanisms of antipsychotics adjusting by epigenetic factors.

A comprehensive summary of questions concerning neurogenic actions of antipsychotic drugs is given by Newton and Duman [122]. 

## 7. From the Bench: Interplay of Schizophrenia, Neuroleptics, and Neurogenesis

Several pathophysiological models have been proposed to explain schizophrenia and may appear to reflect distinct aspects of this disease. None of the pharmacological, genetic, and neurodevelopmental models have been evaluated in detail for translational relevance or to satisfy requirements of the different levels of validity (face, construct, and predictive validity; for review see [123]). Pharmacological models focused on alterations in the dopaminergic, glutamatergic, serotonergic, and GABAergic neurotransmitter systems [54, 124, 125]. They are based on alterations in these neurotransmitter systems, mimicking the *in vivo* conditions which are clinically relevant for schizophrenia. These alterations may be manipulated by drug challenges. 

## 8. First Lesson: The Aspect of Maternal Vitamin D Deficiency

Given the apparent polygenic nature of schizophrenia and the limited translational significance of the available pharmacological models, neurodevelopmental models may offer a better chance of success [126]. Different animal models in schizophrenia research were developed to shed light on the developmental aspects of the disease. 

In the course of ontogenesis there are two critical phases when the organism is susceptible to disturbances which can contribute to schizophrenia, that is, the embryonic/postdelivery phase (first hit) and during puberty (second hit). The vitamin D deficiency model appears to be useful to study the impact of brain disturbances during embryonic/fetal development [127, 128]. Interestingly, some of the vitamin D deficiency effects could be related to later gestational periods thus possibly expanding the hazardous time window for the neurobiological development of schizophrenia [129]. Moreover, it was shown that the neurosteroid can impact brain development by affecting migration and survival of developing neurones in the brain, by influencing brain levels of neurotrophins and their receptors [130], by altering brain apoptotic activity [131], and by exerting immunoregulatory and neuroprotective effects (for review see [127]).

The vitamin D deficiency model is one of the most commonly explored and used also in our lab [29, 132]. Generally, animals from a normal diet control group which were left untreated revealed a basal level of 5-bromo-2′-deoxyuridine (BrdU) immunolabelling in the hippocampal subgranular zone which was in line with previous reports [133]. Cell typing 5 days after BrdU application offered a large pool of BrdU-labelled cells costained with DCX, a marker for immature neurons (~55%). A colabelling with NeuN, a marker for mature neurons, was only rarely found. The second largest group of BrdU-positive cells (~10%) were round or oval, medium-sized, and immunopositive for nestin, an intermediate filament that is expressed in neuronal stem or progenitor cells, identifying these cells as granule cell precursors. The remaining BrdU-positive cells expressed the common astroglia marker GFAP (~4%), the NG2 proteoglycan, a marker for oligodendroglial precursor cells and/or synantocytes (~4%), or were free of any co-labelling (~7%). There were no obvious differences in the distribution of BrdU-immunoreactive cells at different longitudinal levels of the dentate gyrus. 

When cell typing was done 3 weeks after the last BrdU application, about 75% of the BrdU-labelled cells could be identified as granule cells. They had a small round soma, were immunopositive for NeuN, and some of them were shifted from the subgranular cell layer towards the middle part of the granule cell layer. Now, DCX co-labelling was found to be poor. Saline treatment did not alter this BrdU-labelling/costaining pattern. 

Prenatal vitamin D deficiency reduced cell proliferation in the subgranular zone. The loss was proportionally distributed between the different cell types. Due to the very little counts of BrdU-positive cells marked with all nonneuronal markers, only the loss of DCX-positive cells was numerically evident. 

In control animals, the typical (first-generation) neuroleptic haloperidol significantly increased the total number of BrdU-labelled cells. In vitamin D-deficient mice, the deficiency-induced reduction of cell proliferation was completely normalized by haloperidol resulting in a mitotic activity adequate to the untreated control level. In both cases, haloperidol treatment revealed tendentially more DCX-expressing cells. Moreover, a cytoskeletal hypertrophy of radial glia-like GFAP-positive astrocytes, possibly serving as climbing frame for the migrating neuronal newcomers, was found. 

There is general consensus that neuroleptic drugs improve the psychopathology of schizophrenia. Treatment with typical neuroleptics is considered to result in minimal improvement or in worsening of cognitive processes [134], but there are also reports showing that typical neuroleptics provide modest gains in multiple cognitive domains [135]. As hippocampal neurogenesis plays an important role in learning and memory processes [74], we speculate that the previously demonstrated normalization of a vitamin D deficiency-induced habituation deficit in the hole board by haloperidol [136] could result from an at least partially restored mitotic activity. This idea is supported by findings that vitamin D depletion depressed promitotic genes [131, 137]. It is also plausible that there is some kind of exhaustion of the mitotic cell potency due to the overshooting activity in young animals, as it was demonstrated for the vitamin D deficiency model [131] and the NOS knock-out model [138]. Using learning paradigms dependent on hippocampal integrity in subsequent experiments, effects of APDs on both learning behaviour and neurogenesis should be studied in detail. 

## 9. Second Lesson: The Aspect of Maternal Infection

Epidemiological studies have shown that maternal infection and inflammation in definite periods of pregnancy are significantly associated with an increased risk of schizophrenia in the offsprings. Infection with influenza virus [139] or application of polyriboinosinic-polyribocytidylic acid (poly I:C), an inflammatory agent which mimics inflammation by stimulation cytokine release through Toll-like receptor TLR3 activation is accepted models in schizophrenia research (for review see [140]). Prenatal immune stimulation reduces hippocampal neurogenesis [141–144]. The beneficial effects of atypical neuroleptic drugs (APDs), on the other hand, have been attributed to their capacity to increase neurogenesis [22, 145–147]. Together with the group of Weiner and Piontkewitz [28], some of the present authors studied the effects of adolescent poly I:C and risperidone treatment by analyzing a battery of cellular markers referring to cell proliferation and differentiation of hippocampal cell populations. The offspring of poly I:C-treated dams were characterized by an impaired neurogenesis including a decrease of calretinin-positive neurons, disturbed microvascularization and granular cell density in the dentate area, and a reduction of parvalbumin-expressing interneurons, whose deficit is a well-replicated neuropathological finding in schizophrenia [148]. Risperidone normalized the disturbed cell proliferation and/or survival, the number of calretinin and parvalbumin-expressing cells, and counteracted the disturbance in angiogenesis. 

Together with previous reports on deficient hippocampal neurogenesis in offspring of poly I:C-exposed mice [64, 144] and LPS-exposed rats [141], our findings confirm the hypothesis that impaired neurogenesis is an important aetiopathological factor for hippocampal abnormalities and related cognitive dysfunctions in animal models and in patients with schizophrenia [22, 30, 74, 123, 141, 145–147, 149, 150]. Studies concerning an influence of antipsychotics in the poly I:C model are rare. Thus, Meyer et al. [64] demonstrated that chronic clozapine treatment had no effect on poly I:C-hampered neurogenesis.

With respect to neurogenesis, our findings about a risperidone-mediated normalization of the byprenatal poly I:C disturbed angiogenesis are of special interest. In general, angiogenesis and neurogenesis are closely linked with each other [151]. Thus, VEGF modulates neurogenesis directly and also subsequently releasing neurotrophic factors such as BDNF [145, 152, 153]. Moreover, in a previous study we showed that administration of risperidone was able to increase VEGF expression [30] and angiogenesis [28] in the hippocampus of rats. Given that alterations of brain capillaries have been observed in schizophrenia [154], the demonstrated angiogenic effect of risperidone might be a partial mechanism by which antipsychotics realized their action. 

Deficit of parvalbumin-expressing interneurons is an accepted feature in schizophrenia [148, 155], also demonstrable in animal models [156–160]. Our findings that risperidone counteracts this induced by prenatal poly I:C deficiency may also have important implications for understanding its antipsychotic mechanism. 

To fully complete the present section, it is important to note that, in line with previously reported findings [161, 162], we found no effects of prenatal poly I:C treatment and/or risperidone intervention on astrocytes, oligodendrocytes, and microglial cells. 

## 10. Third Lesson: The Aspect of Imbalances in Central Glutamatergic Neurotransmission

Glutamatergic alterations have been consistently showed in psychosis, since its earliest stages [54]. Repeated administration of noncompetitive NMDA receptor antagonists like ketamine, dizocilpine, and phencyclidine (PCP) to neonatal and pubertal rats leads to a number of molecular, neurochemical, and behavioural alterations that resemble those observed in schizophrenia [156, 163, 164]. Administration of the NMDA receptor antagonist dizocilpine and PCP in late fetal and early postnatal period of life in the rat will increase neuronal death by apoptosis [165]. On the contrary, administration of these substances to rats at an adult age will increase neuronal damage by necrosis with subsequent gliosis [166] which results in enduring alteration in the neuronal circuitry. Maeda and coworkers [66] showed that PCP-induced decreased adult neurogenesis was counteracted by coadministered glycine and D-serine confirming the involvement of NMDA receptors in disruption of neurogenesis. Moreover, they were able to demonstrate a reconstruction of neurogenesis by clozapine, but not haloperidol. 

However, we found that acute application of ketamine in sub-anaesthetic doses had no effect on cell proliferation. Animals, decapitated 3 weeks after ketamine application, however, showed a significant increased number of BrdU-labelled nuclei in the subgranular zone compared to saline-treated and untreated animals, whereby the cell-type assignment did not differ between the groups. There was no difference between the left and the right hippocampus, but significantly more BrdU-labelled cells were found in the lateral than in the medial blade of the dentate gyrus. In our first respective paper [167], this was interpreted as stimulating effect of ketamine on neurogenesis. Later on [30], however, we speculated that the withdrawal rather than the application of ketamine was essential and that beside an increase of cell proliferation there was a better survival of proliferated cells. These effects were accompanied by an enhanced mRNA level of BDNF. 

Haloperidol and the atypical antipsychotic risperidone increased the total number of BrdU-labelled cells surviving for three weeks within the granule cell layer in untreated animals. Hereby, VEGF (vascular endothelial growth factor, signalling protein involved in angiogenesis and cell proliferation in general), MMP2 (matrix metallopeptidase 2 (Gelatinase A)), a proteolytic enzyme involved in cell proliferation, adhesion, and migration), CREB, and p38 MAP kinase seemed to be involved at mRNA as well as protein levels. The ketamine withdrawal-induced changes in proliferation/survival, however, were not additionally affected by the neuroleptics [30]. 

Malberg and Monteggia [168] showed that chronic administration of haloperidol increased the level of BDNF in the frontal cortex and amygdala, a possible mechanism for the neuroproliferative potency of haloperidol. Together with its direct effect on MMP2 and the subsequent effect on VEGF (possibly by processing the VEGF binding proteins HARP (heparin affine regulatory peptide)) and CTGF (connective tissue growth factor), the cell proliferative/protective potency of haloperidol is plausible. 

Nevertheless, the demonstrated haloperidol effect on cell proliferation is in agreement with some, but not all, previous reports [24, 25, 27, 169]. The differences can reflect methodological heterogeneity across different experimental settings (dosage, application regime, and used rat strain). 

Interestingly, in the PCP model risperidone was unable to reverse the PCP-induced decreases in parvalbumin expression in the prefrontal cortex [65]. This indicates that the antipsychotic effects of risperidone differ (prenatal poly I:C insult (see above) versus chronic administration of PCP to adult animals). 

## 11. Fourth Lesson: The Aspect of Mechanical Lesions

Lesion models such as the neonatal ventral hippocampal lesion result in schizophrenia-related alterations in behaviour, neurochemistry, and neuropathology when performed on postnatal day (PD) 7, but not on PD 14 or PD 21 [32]. Interestingly, lesions performed in adolescent rats result in less pronounced and qualitatively different schizophrenia-related alterations [170–173]. However, only few data suggesting a link between brain lesion and neurogenesis are available. Lipska et al. [174] and Ashe et al. [175] studied the expression of BDNF mRNA in rats with neonatal lesions of the ventral hippocampus and found consistently a suppressed BDNF level in the dentate gyrus. From that and from the BrdU incorporation studies, they concluded that “a transient disconnection in the CA1 and CA2 area of the hippocampus may have long-lasting consequences for neurogenesis in the dentate gyrus” [176]. Negrete-Díaz et al. [67] showed that nitric oxide (NO) levels in the prefrontal cortex, the occipital cortex, and the cerebellum are higher in the damaged animals and that haloperidol, in part, attenuates these altered NO levels. NO itself is known to be anti-proliferative and it should be allowed to suppose a connection between the enhanced NO level and the reduced BrdU incorporation in animals with ventral hippocampus lesions. It is not clear, however, how haloperidol-induced reduction in NO may lead to a restored cell proliferation. This might be, at least partially, a mechanism by which haloperidol decreased stereotypy in ventral hippocampus damaged rats [177]. 

## 12. Conclusions/Outlook

Clinical and experimental researches indicate that neurogenesis is disturbed in schizophrenia, since its earliest phases. Moreover, antipsychotics specifically interact with these alterations, affecting the neurogenesis. By increasing the neurogenesis it may be possible to provide beneficial gains for processes related with learning and memory formation. The regulation of neurogenesis may be a promising novel target for the treatment and the prevention of schizophrenia. 

## Figures and Tables

**Table 1:** Critical selection of most relevant papers published in the last 13 years.

Author	Year	Finding	Reason for selection
Bayer et al. [1]	1999	Genetic or environmental first hit affects brain development, a second hit later in life initiated the outbreak of schizophrenia	Comprehensive hypothesis concerning schizophrenia etiology

Arnold and Watt [21]	2001	Number of immature cells is increased in the olfactory epithelium of schizophrenics	First report on altered cell density in schizophrenics

Reif et al. [22]	2006	Cell proliferation is diminished in the dentate gyrus of schizophrenics	First report on altered cell proliferation in the human brain

Kippin et al. [23] Wakade et al. [24] Wang et al. [25]	2005 2002 2004	Typical and atypical neuroleptics enhance neurogenesis in the subventricular zone	Effects of neuroleptics on neurogenesis in the subventricular zone

Kodama et al. [26] Wakade et al. [24] Halim et al. [27]	2004 2002 2004	Atypical but not typical neuroleptics interfere with hippocampal neurogenesis	Effects of neuroleptics on neurogenesis in the hippocampus

Piontkewitz et al. [28]	2012	Risperidone partially restored impaired neurogenesis in poly I:C offspring	Effect of an atypical neuroleptic on neurogenesis in an model of maternal infection

Keilhoff et al. [29]	2010	Subchronic treatment with haloperidol ameliorated decreased neurogenesis and normalised behaviour in vitamin D-deficient rats	Effect of neuroleptics on neurogenesis in the vitamin D model

Keilhoff et al. [30]	2010	Risperidone and haloperidol promoted survival in stem cells in the hippocampus of rats subchronically treated with ketamine	Effects of typical and atypical neuroleptics on neurogenesis in the ketamine model

**Table 2:** Survey of the effects of neuroleptics on hippocampal neurogenesis.

Model	Effect on neuroleptics on neurogenesis in the hippocampus	Literature
Vitamin D deficiency	Haloperidol ↑	[29]
Maternal infection	Risperidone ↑	[28]
	Clozapine *Ø*↗	[64]
Ketamine	Haloperidol ↑*	[30]
	Risperidone ↑*	[30]
Phencyclidine	Risperidone *Ø*	[65]
	Clozapine ↑	[66]
Postnatal lesion of ventral hippocampus	Haloperidol *Ø*↗	[67], indirect

Ø: no effect, ↑: increase,  ↗: increase after application of low doses, *: cell survival.
